# ASC and SVF Cells Synergistically Induce Neovascularization in Ischemic Hindlimb Following Cotransplantation

**DOI:** 10.3390/ijms23010185

**Published:** 2021-12-24

**Authors:** Hong Zhe Zhang, Dong-Sik Chae, Sung-Whan Kim

**Affiliations:** 1 Department of Cardiology, College of Medicine, Dong-A University, Busan 49201, Korea; zhanghongzhe@hanmail.net; 2 Department of Orthopedic Surgery, International St. Mary’s Hospital, College of Medicine, Catholic Kwandong University, Incheon 22711, Korea; 3 Department of Medicine, College of Medicine, Catholic Kwandong University, Gangneung 25601, Korea

**Keywords:** angiogenesis, cotransplantation, mesenchymal stem cells, stromal vascular fraction, ischemic hindlimb

## Abstract

Previously, we reported the angio-vasculogenic properties of human stromal vascular fraction (SVF) and adipose tissue-derived mesenchymal stem cells (ASCs). In this study, we investigated whether the combination of ASCs and SVF cells exhibited synergistic angiogenic properties. We conducted quantitative (q)RT-PCR, Matrigel plug, tube formation assays, and in vivo therapeutic assays using an ischemic hind limb mouse model. Immunohistochemical analysis was also conducted. qRT-PCR results revealed that FGF-2 was highly upregulated in ASCs compared with SVF, while PDGF-b and VEGF-A were highly upregulated in SVF. Conditioned medium from mixed cultures of ASCs and SVF (A+S) cells showed higher Matrigel tube formation and endothelial cell proliferation in vitro. A+S cell transplantation into ischemic mouse hind limbs strongly prevented limb loss and augmented blood perfusion compared with SVF cell transplantation. Transplanted A+S cells also showed high capillary density, cell proliferation, angiogenic cytokines, and anti-apoptotic potential in vivo compared with transplanted SVF. Our data indicate that A+S cell transplantation results in synergistic angiogenic therapeutic effects. Accordingly, A+S cell injection could be an alternative therapeutic strategy for treating ischemic diseases.

## 1. Introduction

Since its inception, stem cell therapy has emerged as an attractive therapeutic option for the treatment of damaged organs. Among stem cells, mesenchymal stem cells (MSCs) are advantageous for cell therapy due to their multipotency, easy access, easy isolation and culture from various tissues, and high yield [1,2]. The therapeutic mechanisms of MSCs include differentiation and paracrine effects through the secretion of various growth factors such as fibroblast growth factor (FGF) and epidermal growth factor (EGF) [3,4]. In fact, transplanted MSCs have been shown to be engrafted in blood vessels and the pericytic area, and to play a key role in vascular stabilization [4,5,6].

Adipose-derived stromal vascular fraction (SVF) cells are a heterogeneous cell population comprising mesenchymal progenitor/stem cells, preadipocytes, endothelial cells, pericytes, T cells, and M2 macrophages [7]. Freshly isolated SVF cells are now widely used in cosmetic surgery and other clinical applications [7]. We also previously demonstrated the angiogenic characteristics of SVF and adipose-derived MSCs (ASCs) in ischemic hindlimbs and wounds [8,9]. We found different angiogenic properties of these two cells and tested those synergistic angiogenic effects.

Angiogenic factors such as VEGF-A/PDGF-b and FGF-2 show potent synergistic effects in promoting neovascularization [10,11]. Dual stem cell therapy, such as early/late endothelial progenitor cells or cardiomyocytes derived from induced pluripotent stem cells, also synergistically improves cardiac function and vascular regeneration in the ischemic heart by stem cell homing and the prolonged secretion of paracrine factors [12,13].

Over the past ten years, we attempted to find the best stem cell lineage or combination to enhance the therapeutic potential in damaged tissues [14]. Stem cell therapy has been limited by marginal or transitional influences in ischemic heart diseases [15,16]. In this study, we investigated whether ASCs and SVF cells exhibit synergistic therapeutic potential in ischemic hindlimbs following their cotransplantation.

## 2. Results

### 2.1. Cell Characteristics and Angiogenic Properties of ASCs and SVF Cells

**Figure 1:**
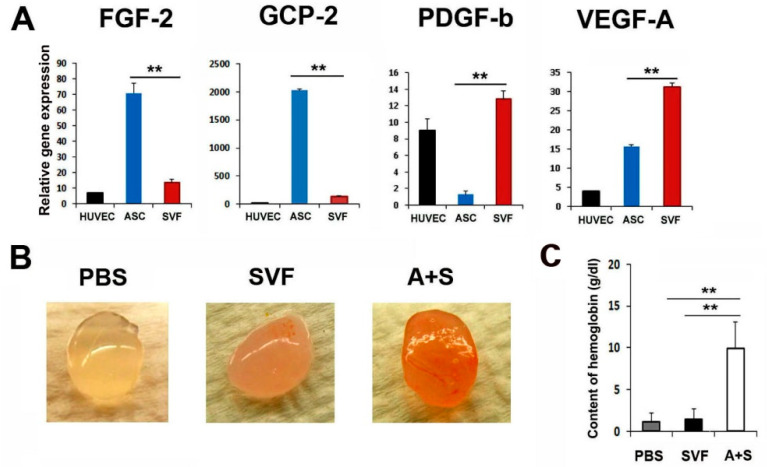
Angiogenic properties of ASCs and SVF. (**A**) Comparison of pro-angiogenic gene expressions. The expression levels of angiogenic genes were examined by qRT-PCR. All values were normalized to GAPDH. ** *p* < 0.01; *n* = 5 per group. (**B**) In vivo Matrigel plug assay. Representative photograph of Matrigel plug injected with SVF cells or A+S cells at two weeks after cell injection. (**C**) Quantification of hemoglobin content. ** *p* < 0.01; *n* = 4 per group.

To characterize the angiogenic properties of ASCs and SVF cells, we performed qRT-PCR and enzyme-linked immunosorbent assay. Interestingly, ASCs expressed higher levels of FGF-2 compared to SVF cells (Figure 1A and Supplementary Figure S1). SVF cells expressed higher levels of PDGF-b and VEGF-A compared to ASCs. Thus, we hypothesized that these cells could exhibit synergistic angiogenic potential when mixed and transplanted. 

Briefly, to examine the in vivo vasculogenic potential of the mixture of ASCs and SVF cells (A+S), a Matrigel plug assay was performed. SVF or A+S cells mixed with 500 μL Matrigel were transplanted subcutaneously into nude mice. Matrigel plugs were harvested 2 weeks later, and the hemoglobin content was examined. Interestingly, Matrigel plugs containing A+S cells were highly filled with red blood cells compared with plugs containing SVF cells only, suggesting the formation of a functional vasculature in the presence of A+S (Figure 1B,C). However, no red blood cells were observed in the PBS control group.

### 2.2. Culture Media of A+S Show In Vitro Vasculogenic Potential

**Figure 2:**
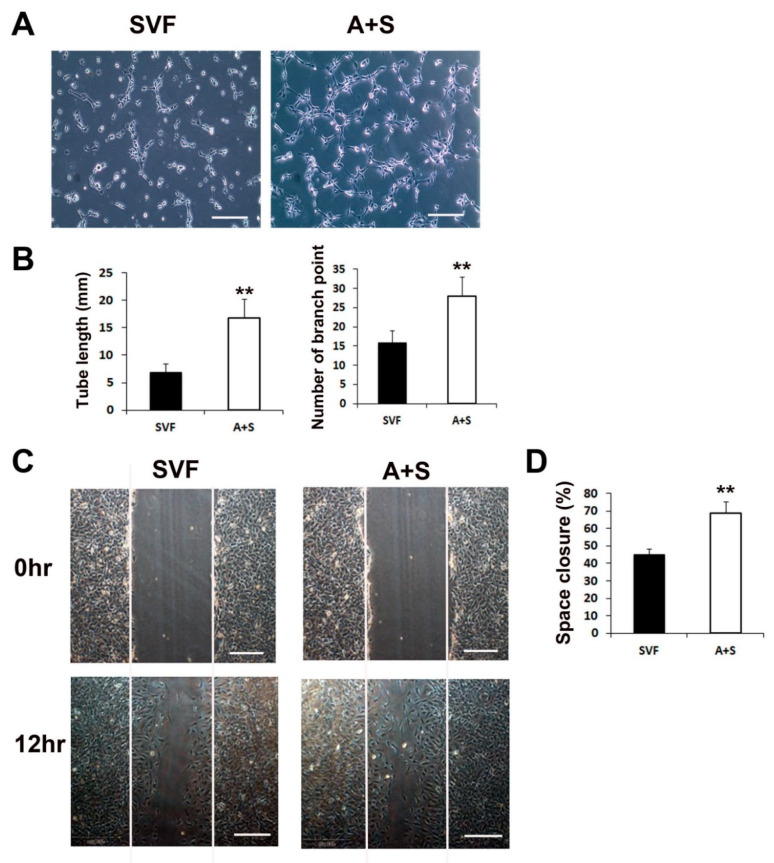
Matrigel tube formation and scratch wound assays. (**A**) Representative images of tube formation after incubation with CM of SVF or A+S cells. Bars, 100 μm. (**B**) The tube length and branch point numbers were significantly increased in the culture with CM from A+S cells compared with those in the culture with CM from SVF cells. ** *p* < 0.01; *n* = 5 per group. (**C**) Representative photograph scratch wound closure by HUVECs after incubation with CM. Bars, 200 μm. (**D**) The in vitro wound closure assay revealed that A+S cell CM highly improved wound closure by HUVECs compared with the SVF CM. ** *p* < 0.01; *n* = 5 per group.

We performed a Matrigel tube formation assay and scratch wound closure assay to test the in vitro vasculogenic and cell migration potential. ASCs and SVF cells (1 × 10^6^ cells each) were seeded into T-75 cell culture flasks and grown in LG-DMEM containing 10% FBS for 5 days. Culture media (CM) from each sample was collected and used to examine its vasculogenic potential. The results revealed that the CM of A+S cells induced significantly higher tube lengths and branching points than the CM of SVF cells (Figure 2A,B). In addition, to investigate cell migration during wound closure, a scratch wound closure assay was performed. The results showed that the A+S cell CM significantly increased the rate of HUVEC wound closure compared to that of SVF cells (Figure 2C,D). 

### 2.3. A+S Exhibits High Cytoprotective Effects 

**Figure 3:**
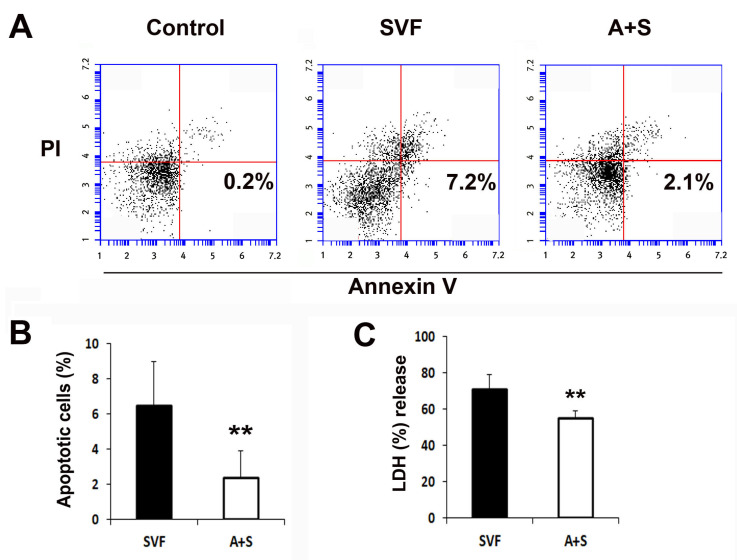
In vitro anti-apoptosis assay. (**A**) Representative photograph of apoptosis assay. Apoptosis was analyzed by fluorescent-activated cell sorter (FACS). (**B**) The levels of annexin V+ and propidium iodide (PI)- cells were significantly lower in A+S than in SVF cells. *n* = 5 per group. (**C**) Comparison of LDH secretion into the culture supernatant after treatment with H_2_O_2_. *n* = 5 per group. ** *p* < 0.01.

To evaluate the cytoprotective effects of the cells, 1 × 10^6^ SVF cells and A (0.5 × 10^6^) + S (0.5 × 10^6^) cells were plated and treated with camptothecin or H_2_O_2_, and the rate of apoptotic cells and the levels of lactate dehydrogenase (LDH) were measured. The rate of apoptotic cells in the A+S group was significantly lower than that in the SVF cells, as determined by flow cytometry (Figure 3A,B). Similarly, A+S were significantly protected against H_2_O_2_ (Figure 3C). 

### 2.4. Transplantation of A+S Cells Exhibits High Therapeutic Effects on Hind Limb Ischemia 

**Figure 4:**
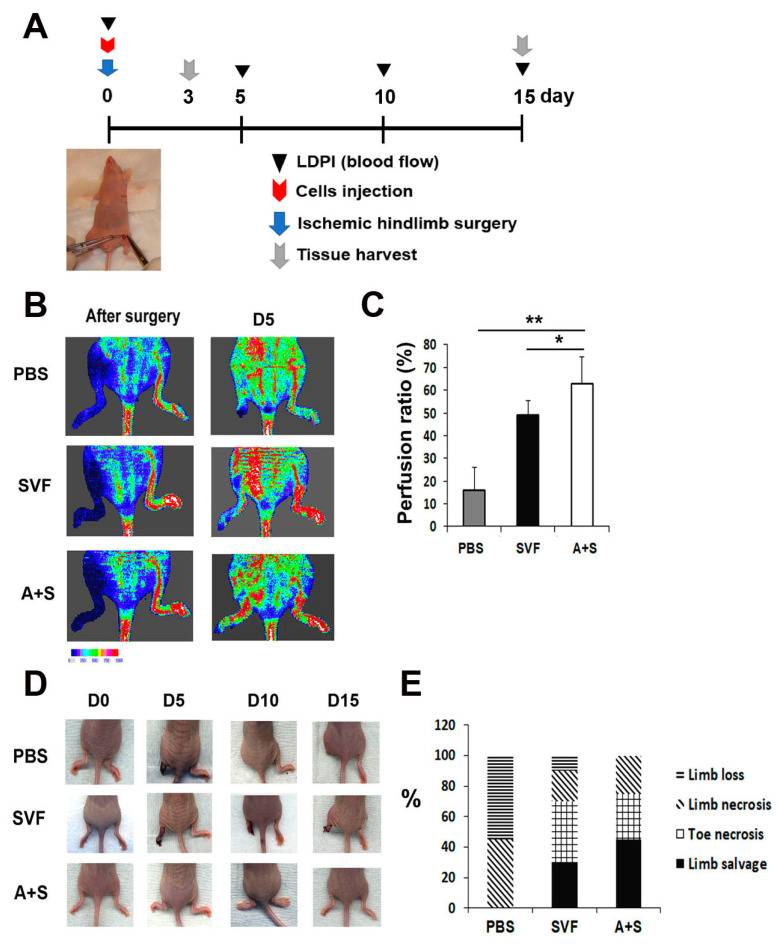
Analysis of therapeutic effects in a hind limb ischemia model after cell injection. (**A**) Schematic representation of the procedure for the hind limb ischemia surgery, cell injection, laser doppler blood perfusion (LDPI) analysis and the collection of tissues. (**B**) Representative LDPI images. The recovery of blood flow in ischemic hind limbs was measured. (**C**) Quantitative analysis of blood perfusion 2 weeks after cell injection. *n* = 7 per group. ** *p* < 0.01; * *p* < 0.05. (**D**) Representative pictures of ischemic hind limb salvage after cell injection. (**E**) Quantitative analysis of limb salvage after cell injection.

To investigate the therapeutic potential of A+S cells, hindlimb ischemia was induced in nude mice. SVF cells, A+S cells, and PBS (control group) were injected intramuscularly into the hind limb. The rate of blood perfusion was analyzed using laser Doppler perfusion. Blood perfusion was significantly increased on day 5 in the limbs injected with A+S cells compared to the limbs injected with SVF cells or PBS (Figure 4A,B). In addition, the A+S cell treated limbs showed a higher limb salvage ratio than those injected with SVF cells and PBS (Figure 4C,D).

### 2.5. Transplantation of A+S Enhanced Capillary Density in Ischemic Hindlimb 

**Figure 5:**
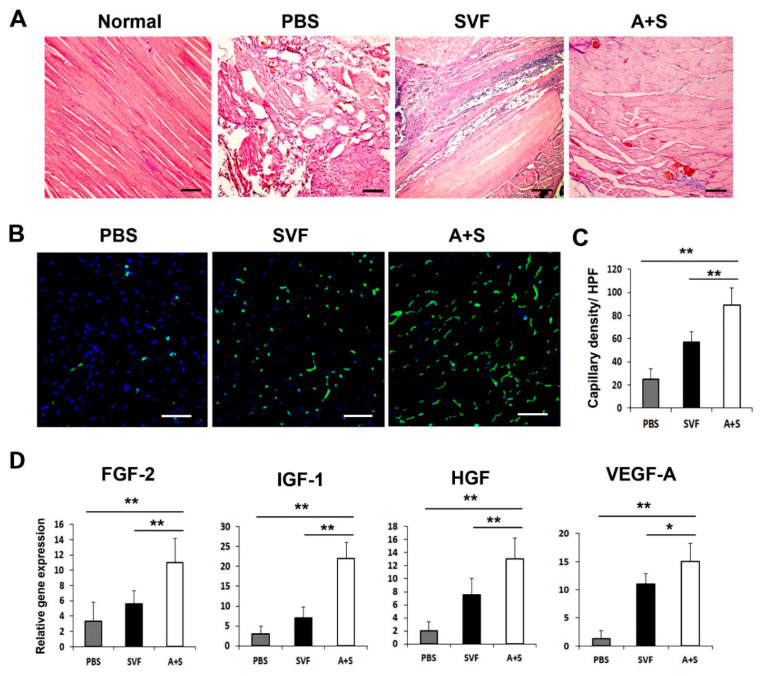
Histological analysis and angiogenic factors expression in hind limb tissue. (**A**) Histological images of H&E-stained hind limb tissues at 15 days after cell injection. Bars = 200 μm. (**B**) Representative pictures of capillary density in hindlimb tissues at 4 weeks after cell injection. Nuclear DAPI staining is blue; ILB4 staining is green. Bars = 50 μm (**C**) Analysis of capillary density in hindlimb tissues after cell injection. ** *p* < 0.01; * *p* < 0.05; *n* = 5 per group. (**D**) Analysis of angiogenic factors expression in hindlimb tissue three days after cell transplantation. ** *p* < 0.01; * *p* < 0.05; *n* = 5 per group. Abbreviation: High-power field (HPF).

To elucidate the mechanism underlying the enhanced therapeutic effects of A+S cell treatment, we performed H&E staining and examined the capillary density of the hind limb tissues using isolectin B4 (ILB4) as a marker for endothelial cells. The results revealed decreased muscle degeneration in the A+S injected group compared to that in the PBS or SVF cell-injected group (Figure 5A). Capillary density was significantly higher in the A+S injected group than in the PBS or SVF cell injected group (Figure 5B,C). qRT-PCR and western blot analysis were also performed on the hind limb tissues. As expected, A+S cell transplantation significantly upregulated FGF-2, IGF-1, HGF, and VEGF-A compared to the SVF cell transplanted hind limb tissues at three days after cell transplantation (Figure 5D). These results indicate that A+S cell injection promoted the expression of multiple angiogenic factors for vascular regeneration. 

## 3. Discussion

Although stem cell therapy has been an attractive tool in the tissue regenerative field, there are still limitations such as marginal or transitional therapeutic effects. In this study, we first demonstrated that human adipose tissue-derived ASCs and SVF cells synergistically promoted neovasculogenesis following their cotransplantation. This synergism was confirmed in in vitro and in vivo experiments. 

Stem cell transplantation has been attempted also in peripheral artery disease with uncertain results [17]. The mobilization of stem cells has been also proposed as a mediator of beneficial physical activity effects [18]. Although stem cells have promising therapeutic potential, there are still controversies regarding the minimal therapeutic effects in ischemic heart [19]. In the past decade, we have attempted to develop an enhanced therapeutic strategy for cell-based therapy. Recently, we reported the attractive features of human adipose tissue-derived SVF cells as a stem cell source due to the abundant number of stem cells and their high angio-vasculogenic potential [8]. To promote the therapeutic effects of SVF cells, we explored combination therapy using ASCs, which is a popular adult stem cell source. In this study, we found distinct angiogenic properties of ASCs and SVF cells, and we hypothesized that therapy with the combination of these two types of cells could have synergistic therapeutic effects in ischemia. Similarly, early endothelial progenitor cells and outgrowth endothelial cells derived from human peripheral blood have different functions in neovascularization, and transplantation with a mixture of the two types of cells showed synergistic neovascularization through cytokines [20]. In addition, dual stem cell therapy has recently shown regenerative effects or synergistically improved stem cell homing, cardiac function/remodeling, and vascular regeneration following myocardial infarction [12,13]. Thus, these attractive features of the two cell populations as stem cell sources for cell therapy prompted us to study their potential to contribute to the regeneration of ischemic tissues.

We found that ASCs and SVF cells secrete different proangiogenic factors. ASCs express high levels of FGF-2, whereas SVF cells express high levels of VEGF-A and PDGF-b. Thus, we hypothesized that a mixture of these two cell types could show synergistic neovasculogenic effects in ischemic tissues. In fact, combination therapy with VEGF-A and FGF-2 showed potent synergistic effects on neovascular formation [21]. In addition, the combination of VEGF-A and PDGF-b or FGF-2 and PDGF-b also exhibited potent synergistic effects in promoting neovascularization [10,11]. In line with these reports, CM derived from the coculture of ASCs and SVF cells resulted in an enhanced tube network formation and cell migration, suggesting synergism between them. 

PDGF-b is also associated with angiogenesis, regulates vessel growth, and increases the survival and proliferation of endothelial cells [22]. FGF-2 inhibits endothelial cell apoptosis through Bcl-2-dependent and independent mechanisms [23]. In addition, FGF-2 binds to fibrin and supports prolonged endothelial cell growth [24]. VEGF is a key regulator of vessel growth and regression and acts as an endothelial survival factor by protecting endothelial cells from apoptosis [25]. The mechanism of the synergistic effect of FGF-2 and VEGF-A has also been elucidated [26]. These two factors synergically enhance endogenous PDGF-b/PDGFR signaling to promote mature blood vessel formation [26]. The signaling axis of PDGF-BKLF4/VEGF contributes to vascular remodeling [27].Thus, therapy with the combination of cells expressing VEGF-A, PDGF, and FGF-2 could exhibit an outstanding advantage in increasing angiogenesis. Our data also demonstrated that the upregulation of synergistic angiogenic factors elicited therapeutic responses that enhanced tube network formation and cell proliferation/migration.

In vivo cell survival in an ischemic environment is important to overcoming the marginal therapeutic effects of stem cells. In fact, the poor cell engraftment and low survival potential of stem cells are one of the main limitations regarding their therapeutic applications [28].

Our data demonstrated that a mixture of A+S cells has a high anti-apoptotic capacity compared to single SVF cells. These results might be associated with the high levels of FGF expression in ASCs. FGF-2 enhances the survival of a variety of endothelial, epithelial, and hematopoietic cell lines after injury [29]. Our data also showed that dual stem cell injection induced high cell proliferation and had anti-apoptotic effects in tissues. It is likely that the paracrine secretion of FGF-2 affects the proliferation and survival of cells in ischemic muscle tissues. The mechanisms underlying the protective effects of FGF-2 have been previously reported. FGF-2 increases the breakdown of reactive oxygen or causes cell-cycle arrest for repair and enhances the injury–repair mechanism [29].

Cell transplantation studies have confirmed these synergistic therapeutic results in vivo. Dual cell transplantation induced higher blood perfusion and increased levels of multiple angiogenic factors in ischemic tissues. These data suggest that the major therapeutic mechanism of dual cell transplantation involves paracrine secretion. The rate of endothelial transdifferentiated cells might be too low to account for the synergistic therapeutic effects such as neovascularization in ischemic tissue. However, an investigation in the rate of engraftment or the transdifferentiation of injected cells in vivo for a long period is needed. 

In conclusion, two different types of cells can be obtained from adipose tissues, and the paracrine secretion of different cytokines by these cells may play important roles in enhancing neovascularization. Thus, these mixed cells synergistically increase angiogenesis in hindlimb ischemic tissue following their cotransplantation. The transplantation with mixtures of cells could be an alternative strategy to enhance the therapeutic effects of cell-based therapy in the future. 

## 4. Materials and Methods

### 4.1. SVF Isolation

Human SVF cells were isolated from the subcutaneous fat tissue after receiving informed consent from healthy donors using the guidelines approved by the institutional review boards of Catholic Kwandong University Hospital. The SVF cells of three donors were separated using a previously described method [30]. Briefly, the fat tissue was digested with 0.075% collagenase solution for 1 h at 37 °C and then separated by centrifugation. Cell pellet was resuspended in red blood cell lysis buffer and the suspended cells were filtered using 100-μm mesh filter (BD, San Jose, CA, USA). 

### 4.2. Cell Culture

Human umbilical vein endothelial cells (HUVECs) were purchased from the ATCC (Manassas, VA, USA) and cultured in endothelial growth complete media (EGM-2) (Lonza Walkersville, MD, USA). Human ASCs were isolated and cultured as previously report [31]. In brief, the same three donors of SVF were plated in the culture plates and maintained at 37 °C under 5% CO_2_ in culture medium (a-MEM, 10% fetal bovine serum (FBS), 100 U/mL of penicillin, and 100 mg/mL of streptomycin). After 1 week, adherent ASCs were observed in the culture plates and non-adherent cells were removed and ASCs were expanded with culture medium.

### 4.3. Real-Time Polymerase Chain Reaction (PCR) Analyses

Quantitative real-time (qRT)–PCR assays were performed as previously described [14,32]. Briefly, total RNA was isolated from cells using RNA-stat (Iso-Tex Diagnostics, Friendswood, TX, USA) and a previously reported method [33]. Extracted RNA was subsequently reverse transcribed using Taqman Reverse Transcription Reagents (Applied Biosystems, Foster City, CA, USA), according to the manufacturer’s instructions. The synthesized cDNA was subjected to qRT–PCR using human/mouse-specific primers and probes. RNA levels were quantitatively assessed using an ABI PRISM 7000 Sequence Detection System (Applied Biosystems, Fostercity, CA, USA). Relative mRNA expression normalized to GAPDH expression was calculated. 

### 4.4. qRT-PCR Primers

All primer/probe sets were purchased from Applied Biosystems (Table 1)

### 4.5. Matrigel Plug In Vivo Assay

Matrigel plug assay was conducted as described in previous report [34]. Briefly, 7- to 10-weeks-old nude, male mice (Joongang Laboratory Animal Inc. Seoul, South Korea) weighing 18–22 g were anesthetized with isoflurane (induction: 450 mL air, 4.5% isoflurane, maintenance: 200 mL air, 2.0% isoflurane, Baxter International, Inc., Deerfield, IL, USA) and 2 × 10^5^ cells with Matrigel 500 μL were injected subcutaneously in mice. After 2 weeks, euthanasia was conducted and the Matrigel plugs were harvested. The levels of hemoglobin content were examined by Drabkin’s Reagent Kit (Sigma Aldrich, St. Louis, MO, USA).

### 4.6. Matrigel Tube Formation Assay

Culture media (CM) were collected as previously described with modifications [35]. Briefly, cells (1 × 10^6^) were seeded into T-75 cell culture flasks and grown in low-glucose Dulbecco’s modified Eagle’s medium (LG-DMEM) (Gibco, Grand Island, NY, USA) containing 10% FBS, 100 U/mL penicillin, and 100 mg/mL streptomycin (Gibco) for 48 h until the cells reached an approximate confluence of 90%. Each sample was then centrifuged at 1000× *g* for 5 min, and the supernatants were harvested. To examine the tube formation potential, HUVECs at a concentration of 1 × 10^4^ cells/well were embedded in LG-DMEM containing 1% FBS (control) and each CM derived from SVFs and ASCs in basement membrane matrix gel (Matrigel, BD)-coated glass slides (NUNC). After 5 h of incubation, representative fields were randomly photographed using inverted microscopy, and the tube length and branching point from each sample were measured as previously described method [36]. 

### 4.7. Scratch Wound Assays

Scratch wound assays were conducted per a previously reported study [35]. HUVECs were seeded to a final density of 1 × 10^5^ cells/well in 24-well culture plates and incubated at 37 °C in 5% CO_2_ for 24 h to produce confluent monolayers. Monolayers were scratched using a sterile pipette tip and incubated with CM. To measure cell mobility, we obtained images at six random fields after scratching and CM incubation. Wound areas were measured by the NIH Image program (http://rsb.info.nih.gov/nih-image/ (accessed on 10 November 2021).

### 4.8. Apoptosis Assay 

We induced apoptosis by treating the cells with camptothecin (6 μM, Sigma-Aldrich, St-louis, MO, USA for 4 h. Apoptotic cells were measured using propidium iodide (PI) and an Annexin V-FITC binding assay kit II (BD PharMingen, San Diego, CA, USA), according to the manufacturer’s protocol. The apoptotic cells were analyzed using a FACScan (Becton Dickinson).

### 4.9. Measurement of Lactate Dehydrogenase (LDH)

To examine the cellular changes, cells were placed into serum-free medium and treated with H_2_O_2_ (100 μM) for 60 min. To induce a cellular response similar to that caused by ischemia, H_2_O_2_ was used as an oxidant [10]. The level of LDH in the supernatant was measured by LDH assay kit (Sigma Aldrich).

### 4.10. Induction of Ischemic Hindlimb Model and Cell Transplantation

Experimental protocols were approved by the Catholic Kwandong University Institutional Animal Care and Use Committee. In addition, all procedures were performed in accordance with the Guide for the Care and Use of Laboratory Animals published by the US National Institutes of Health (NIH Publication No. 85-23, revised 2011. Nude, male mice aged 7- to 10-weeks-old (Joongang Laboratory Animal Inc., Seoul, Korea). Animal experiments were performed as described previously [14]. Briefly, mice were anesthetized with isoflurane (induction: 450 mL air, 4.5% isoflurane; maintenance: 200 mL air, 2.0% isoflurane, Baxter International, Inc., Deerfield, IL, USA) and the depth of anesthesia was monitored by respiratory rate and lack of withdrawal reflex upon toe pinching [4]. Hindlimb ischemia was induced by ligation of the right femoral artery. PBS (control group) or solutions of 1 × 10^6^ ASCs or A+S (ASCs; 0.5 × 10^6^, SVF; 0.5 × 10^6^) were prepared in PBS and then intramuscularly injected into the ischemic hindlimb area after surgery (*n* = 7 for each group). Buprenorphine (0.1 mg/kg) was injected subcutaneously at the end of the surgery and every 5 h until they recovered. Euthanasia was conducted by intravenous injection of thiopental sodium (40 mg/kg). To measure serial blood flow, we used a laser Doppler perfusion image (LDPI) analyzer (Moor Instruments, Axminster, UK) [37]. Limb salvage was defined as retention of the limb without the amputation or necrosis of foot and toes. 

### 4.11. Histological Analysis

All histological experiments were conducted as previously described [33,38]. The adductor muscles of hindlimbs were harvested, fixed in 4% paraformaldehyde for 4 h, and incubated overnight in a 15% sucrose solution. The tissues were embedded in OCT compound (Sakura Finetek USA, Torrance, CA, USA), snap frozen in liquid nitrogen, and sectioned in thickness increments of 10–20 μm. For capillary density measurement, six frozen sections from each group of ischemic tissue were stained with biotinylated isolectin B4 primary antibody (anti-ILB4, 1:250; Vector Laboratory Inc., Burlingame, CA, USA) followed by streptavidin Alexa Fluor 488 secondary antibody (1:400; Invitrogen, Carlsbad, CA, USA). Five fields from five tissue sections were randomly selected, and the number of capillaries was counted in each field. Photographs were taken using fluorescent inverted microscopy and confocal microscopy.

### 4.12. Statistical Analysis

All data are presented as mean ± SD. We performed statistical analyses using Student’s *t*-test for comparisons of the two groups, and ANOVA with Bonferroni’s multiple comparison test using SPSS v11.0. Data with *p* < 0.05 were considered statistically significant.

## Figures and Tables

**Table 1:** Information of qRT-PCR primers.

Origin	Name	Primer
Human	Vascular Endothelial Growth Factor (VEGF)-A	Hs99999070_m1
Human	Fibroblast Growth Factor-2 (FGF-2)	Hs00266645_m1
Human	Platelet Derived Growth Factor (PDGF)-b	Hs00966526-m1
Human	GAPDH	Hs99999905_m1
Mouse	FGF-2	Mm00433287_m1
Mouse	Hepatocyte Growth Factor (HGF)	Mm01135184_m1
Mouse	Insulin Growth Factor (IGF)-1	Mm00439560_m1
Mouse	VEGF-A	Mm01204733_m1
Mouse	GAPDH	Mm99999915_g1

## Supplementary Materials

The following supporting information can be downloaded at: https://www.mdpi.com/article/10.3390/ijms23010185/s1.

## References

[1] Nakagami H., Morishita R., Maeda K., Kikuchi Y., Ogihara T., Kaneda Y. (2006). Adipose tissue-derived stromal cells as a novel option for regenerative cell therapy. J. Atheroscler. Thromb..

[2] Zuk P.A., Zhu M., Ashjian P., De Ugarte D.A., Huang J.I., Mizuno H., Alfonso Z.C., Fraser J.K., Benhaim P., Hedrick M.H. (2002). Human adipose tissue is a source of multipotent stem cells. Mol. Biol. Cell.

[3] Kim S.W., Lee D.W., Yu L.H., Zhang H.Z., Kim C.E., Kim J.M., Park T.H., Cha K.S., Seo S.Y., Roh M.S. (2012). Mesenchymal stem cells overexpressing GCP-2 improve heart function through enhanced angiogenic properties in a myocardial infarction model. Cardiovasc. Res..

[4] Kim S.W., Zhang H.Z., Kim C.E., An H.S., Kim J.M., Kim M.H. (2012). Amniotic mesenchymal stem cells have robust angiogenic properties and are effective in treating hindlimb ischaemia. Cardiovasc. Res..

[5] Traktuev D.O., Merfeld-Clauss S., Li J., Kolonin M., Arap W., Pasqualini R., Johnstone B.H., March K.L. (2008). A population of multipotent CD34-positive adipose stromal cells share pericyte and mesenchymal surface markers, reside in a periendothelial location, and stabilize endothelial networks. Circ. Res..

[6] Kim S.W., Zhang H.Z., Kim C.E., Kim J.M., Kim M.H. (2013). Amniotic mesenchymal stem cells with robust chemotactic properties are effective in the treatment of a myocardial infarction model. Int. J. Cardiol..

[7] Han S., Sun H.M., Hwang K.C., Kim S.W. (2015). Adipose-Derived Stromal Vascular Fraction Cells: Update on Clinical Utility and Efficacy. Crit. Rev. Eukaryot. Gene Expr..

[8] Jin E., Chae D.S., Son M., Kim S.W. (2017). Angiogenic characteristics of human stromal vascular fraction in ischemic hindlimb. Int. J. Cardiol..

[9] Chae D.S., Han S., Son M., Kim S.W. (2017). Stromal vascular fraction shows robust wound healing through high chemotactic and epithelialization property. Cytotherapy.

[10] Richardson T.P., Peters M.C., Ennett A.B., Mooney D.J. (2001). Polymeric system for dual growth factor delivery. Nat. Biotechnol..

[11] Cao R., Brakenhielm E., Pawliuk R., Wariaro D., Post M.J., Wahlberg E., Leboulch P., Cao Y. (2003). Angiogenic synergism, vascular stability and improvement of hind-limb ischemia by a combination of PDGF-BB and FGF-2. Nat. Med..

[12] Park S.J., Kim R.Y., Park B.W., Lee S., Choi S.W., Park J.H., Choi J.J., Kim S.W., Jang J., Cho D.W. (2019). Dual stem cell therapy synergistically improves cardiac function and vascular regeneration following myocardial infarction. Nat. Commun..

[13] Theiss H.D., Vallaster M., Rischpler C., Krieg L., Zaruba M.M., Brunner S., Vanchev Y., Fischer R., Grobner M., Huber B. (2011). Dual stem cell therapy after myocardial infarction acts specifically by enhanced homing via the SDF-1/CXCR4 axis. Stem. Cell Res..

[14] Kim S.W., Kim H., Cho H.J., Lee J.U., Levit R., Yoon Y.S. (2010). Human peripheral blood-derived CD31+ cells have robust angiogenic and vasculogenic properties and are effective for treating ischemic vascular disease. J. Am. Coll. Cardiol..

[15] Weil B.R., Manukyan M.C., Herrmann J.L., Wang Y., Abarbanell A.M., Poynter J.A., Meldrum D.R. (2010). Mesenchymal stem cells attenuate myocardial functional depression and reduce systemic and myocardial inflammation during endotoxemia. Surgery.

[16] Wu J., Li J., Zhang N., Zhang C. (2011). Stem cell-based therapies in ischemic heart diseases: A focus on aspects of microcirculation and inflammation. Basic Res. Cardiol..

[17] Biscetti F., Bonadia N., Nardella E., Cecchini A.L., Landolfi R., Flex A. (2019). The Role of the Stem Cells Therapy in the Peripheral Artery Disease. Int. J. Mol. Sci..

[18] Pasqualini L., Bagaglia F., Ministrini S., Frangione M.R., Leli C., Siepi D., Lombardini R., Marini E., Naeimi Kararoudi M., Piratinskiy A. (2021). Effects of structured home-based exercise training on circulating endothelial progenitor cells and endothelial function in patients with intermittent claudication. Vasc. Med..

[19] Shake J.G., Gruber P.J., Baumgartner W.A., Senechal G., Meyers J., Redmond J.M., Pittenger M.F., Martin B.J. (2002). Mesenchymal stem cell implantation in a swine myocardial infarct model: Engraftment and functional effects. Ann. Thorac. Surg..

[20] Yoon C.H., Hur J., Park K.W., Kim J.H., Lee C.S., Oh I.Y., Kim T.Y., Cho H.J., Kang H.J., Chae I.H. (2005). Synergistic neovascularization by mixed transplantation of early endothelial progenitor cells and late outgrowth endothelial cells: The role of angiogenic cytokines and matrix metalloproteinases. Circulation.

[21] Asahara T., Bauters C., Zheng L.P., Takeshita S., Bunting S., Ferrara N., Symes J.F., Isner J.M. (1995). Synergistic effect of vascular endothelial growth factor and basic fibroblast growth factor on angiogenesis in vivo. Circulation.

[22] Battegay E.J., Rupp J., Iruela-Arispe L., Sage E.H., Pech M. (1994). PDGF-BB modulates endothelial proliferation and angiogenesis in vitro via PDGF beta-receptors. J. Cell Biol..

[23] Karsan A., Yee E., Poirier G.G., Zhou P., Craig R., Harlan J.M. (1997). Fibroblast growth factor-2 inhibits endothelial cell apoptosis by Bcl-2-dependent and independent mechanisms. Am. J. Pathol..

[24] Sahni A., Altland O.D., Francis C.W. (2003). FGF-2 but not FGF-1 binds fibrin and supports prolonged endothelial cell growth. J. Thromb. Haemost. JTH.

[25] Grosjean J., Kiriakidis S., Reilly K., Feldmann M., Paleolog E. (2006). Vascular endothelial growth factor signalling in endothelial cell survival: A role for NFkappaB. Biochem. Biophys. Res. Commun..

[26] Kano M.R., Morishita Y., Iwata C., Iwasaka S., Watabe T., Ouchi Y., Miyazono K., Miyazawa K. (2005). VEGF-A and FGF-2 synergistically promote neoangiogenesis through enhancement of endogenous PDGF-B-PDGFRbeta signaling. J. Cell Sci..

[27] Liang S., Yu H., Chen X., Shen T., Cui Z., Si G., Zhang J., Cheng Y., Jia S., Song S. (2017). PDGF-BB/KLF4/VEGF Signaling Axis in Pulmonary Artery Endothelial Cell Angiogenesis. Cell. Physiol. Biochem..

[28] Wu K.H., Mo X.M., Han Z.C., Zhou B. (2011). Stem cell engraftment and survival in the ischemic heart. Ann. Thorac. Surg..

[29] Houchen C.W., George R.J., Sturmoski M.A., Cohn S.M. (1999). FGF-2 enhances intestinal stem cell survival and its expression is induced after radiation injury. Am. J. Physiol..

[30] Zuk P.A., Zhu M., Mizuno H., Huang J., Futrell J.W., Katz A.J., Benhaim P., Lorenz H.P., Hedrick M.H. (2001). Multilineage cells from human adipose tissue: Implications for cell-based therapies. Tissue Eng..

[31] Wagner W., Wein F., Seckinger A., Frankhauser M., Wirkner U., Krause U., Blake J., Schwager C., Eckstein V., Ansorge W. (2005). Comparative characteristics of mesenchymal stem cells from human bone marrow, adipose tissue, and umbilical cord blood. Exp. Hematol..

[32] Chae D.S., Han S., Lee M.K., Kim S.W. (2021). Genome Edited Sirt1-Overexpressing Human Mesenchymal Stem Cells Exhibit Therapeutic Effects in Treating Collagen-Induced Arthritis. Mol. Cells.

[33] Kim S.W., Houge M., Brown M., Davis M.E., Yoon Y.S. (2014). Cultured human bone marrow-derived CD31(+) cells are effective for cardiac and vascular repair through enhanced angiogenic, adhesion, and anti-inflammatory effects. J. Am. Coll. Cardiol..

[34] Fang J., Ding M., Yang L., Liu L.Z., Jiang B.H. (2007). PI3K/PTEN/AKT signaling regulates prostate tumor angiogenesis. Cell. Signal..

[35] Kim S.W., Zhang H.Z., Guo L., Kim J.M., Kim M.H. (2012). Amniotic mesenchymal stem cells enhance wound healing in diabetic NOD/SCID mice through high angiogenic and engraftment capabilities. PLoS ONE.

[36] Kim M.H., Guo L., Kim H.S., Kim S.W. (2014). Characteristics of circulating CD31(+) cells from patients with coronary artery disease. J. Cell Mol. Med..

[37] Kim M.H., Zhang H.Z., Kim S.W. (2011). Combined growth factors enhanced angiogenic potential of cord blood-derived mononuclear cells transplanted to ischemic limbs. J. Mol. Cell. Cardiol..

[38] Kim M.H., Jin E., Zhang H.Z., Kim S.W. (2013). Robust angiogenic properties of cultured human peripheral blood-derived CD31(+) cells. Int. J. Cardiol..

