# Supplementary material for: ASC and SVF Cells Synergistically Induce Neovascularization in Ischemic Hindlimb Following Cotransplantation
Source: Int J Mol Sci. 2021 Dec 24;23(1):185. doi: 10.3390/ijms23010185 (PMC8745515; doi:10.3390/ijms23010185)
Supplement: Supplementary file 1 [file ijms-23-00185-s001.zip › ijms-1494116-supplementary.pdf]

Supplementary materials and method

Enzyme-linked immunosorbent assay (ELISA)

ELISA in vitro FGF-2 and VEGF-A secretion levels were assessed by using ELISA kit (Abcam, Cambridge, MA, USA) per supplier's protocols. Culture mediums (CMs) were collected by method as previously described [1]. In brief, cells were seeded into T-75 cell culture flasks and grown in normal medium or low-glucose DMEM (Gibco) containing 10% FBS, 100 U/mL penicillin and 100 mg/ mL streptomycin (Gibco) for 48 h until the cells reached approximately 80% confluence. Culture media from each sample was then centrifuged for 10 min and the supernatants were collected and used as CM for the study.

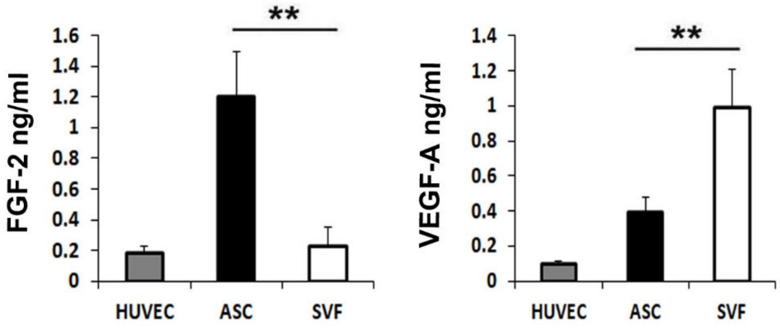

Supplementary Figure S1. Analysis of angiogenic protein levels by ELISA in ASCs and SVF cells. $^{**}p < 0.01$; $n = 5$ per group.

References

[1] Kim SW, Zhang HZ, Guo L, Kim JM, Kim MH. Amniotic mesenchymal stem cells enhance wound healing in diabetic NOD/SCID mice through high angiogenic and engraftment capabilities. PloS one. 2012;7:e41105.
